# Capturing the impact of cultural differences in residency

**DOI:** 10.1186/s12909-021-02548-4

**Published:** 2021-02-18

**Authors:** Douglas Archibald, Alison Eyre, Dorota Szczepanik, Joseph K. Burns, Lionel Laroche

**Affiliations:** 1 Department of Family Medicine, University of Ottawa, Ottawa, Ontario Canada; 2 Bruyère Research Institute, Ottawa, Ontario Canada; 3 MultiCultural Business Solutions Inc., Markham, Ontario Canada

**Keywords:** International medical education, Education environment, Professionalism

## Abstract

**Background:**

Postgraduate training is a period in which residents develop both their medical competency and their professional identity in an environment of apprenticeship. As situated learning suggests, a critical dimension of such apprenticeship is the mode through which trainees can legitimately participate in the practice before they become experts, in this case physicians. One source of barriers to participation is cultural difference between learner and the clinical environment.

**Objective:**

To assess the extent cultural differences create barriers for residents, particularly but not exclusively for international medical graduates (IMGs).

**Methods:**

In 2014–15 a questionnaire was developed with subscales assessing areas such as sense of hierarchy, individuality versus teamwork, and risk tolerance. We refined the instrument by subjecting it to a review panel of experts in postgraduate education followed by “think aloud” sessions with residents.

**Results:**

Piloting this instrument yielded a Cronbach’s alpha of 0.675. When administered to a larger group of residents and faculty representing many specialties, the Impact of Cultural Differences on Residency Experiences (ICDRE) questionnaire revealed a few items for which the Canadian Medical Graduates and International Medical Graduates differed in their mean opinion. The groups were not substantially different overall, but we did observe an interesting diversity of cultural beliefs within each group.

**Conclusions:**

We suggest that the ICDRE may be useful in identifying beliefs which may present challenges to an individual resident or in capturing trends in a resident population so that a specialty program can address the trends proactively. The instrument also provides language with which to anchor preceptors’ evaluations of residents’ professionalism and may serve as an interventional coaching tool.

**Supplementary Information:**

The online version contains supplementary material available at 10.1186/s12909-021-02548-4.

## Background

Supervisors of medical trainees often hesitate to report the shortcomings of their learners during evaluations [1]. This widespread hesitancy appears to be due in part to unclear evaluation criteria [2]; supervisors may identify poor performers by gestalt, but struggle to find words to articulate why a trainee needs remediation. Behaviors associated with communication, collaboration and professionalism are based on assumptions, values, and beliefs, leading to difficulty in describing and teaching them. Additionally, cultural differences can contribute to these behaviours [3–7]. Thus, remediating behaviours related to collaboration and professionalism is challenging, particularly in a context involving International Medical Graduates (IMGs) [8, 9].

Canadian residency programs have many residents who have done their medical school training outside of Canada [10]. For reasons of immigration, recruitment and, in some cases, a desire to return home to Canada, many physicians trained in other countries enter postgraduate medical education in Canada [11]. There are benefits and also challenges presented by this cultural diversity in the Canadian medical profession [12].

The expanding ethnic, cultural and gender diversity of the physician population has been shown to benefit patient care [13–16]. Physicians who are part of an underrepresented or marginalized group are more likely to provide care for underserved groups in their own practice [13, 15]. This synergizes well with the findings of Cooper-Patrick et al. [16], which suggest that many patients form a more participatory therapeutic partnership with physicians of their own ethnicity [16]. In situations where underserved populations represent a particular cultural or ethnic group, greater representation of that group among physicians is likely to enhance the care and satisfaction among those populations.

However, IMGs face a host of challenges, including adapting to a new culture and working in a different medical environment. Numerous studies have shown that IMGs have more difficulties during their medical training, require more remediation and have a lower pass rate on their national licensing examinations than Canadian-trained medical graduates [17–19].

An improved understanding of the impact of different cultures may help trainees to transition and operate effectively within the Canadian medical system. Recent data suggest that over one quarter of practicing physicians in Canada are IMGs [20], the majority of whom work in primary care. Medical graduates immigrate to Canada from all over the world, from regions including the Middle East, Asia, Africa, and Eastern Europe; this results in considerable variation in medical training, clinical experiences, interpersonal competencies and cultural perceptions. Similar issues occur for IMGs (referred to as Foreign Medical Graduates or FMGs) in the United States [21]. An interesting subset of IMGs is Canadians Studying Abroad (CSAs). These are Canadian citizens who grew up in Canada and then attended medical school in another country. The general cultural beliefs of these graduates can be expected to be similar to those of other Canadian citizens, but their experience of medical culture may have been shaped by the country in which they were trained.

To better understand cultural beliefs in our resident population that might constitute challenges in training and to practice, and to establish codified language to describe such challenges, we developed a questionnaire entitled “Impact of Cultural Differences on Residency Experiences” (ICDRE). Culture has many aspects. The country and region where someone is raised influences their cultural norms as does the culture of their family. Institutions also have cultures. Medical cultures vary around the world. It is in medical school that we are introduced to the medical cultural norms of where we are trained. The questionnaire is intended to help all residents, both IMGs and Canadian Medical Graduates (CMGs), to understand the impact of cultural differences on their residency training experiences. It also provides useful language to describe professional qualities with which medical trainees may be struggling and could thereby assist with evaluation of residents.

Many studies have shown that certain issues, such as communication skills and expectations of training, are more common in IMG residents and need to be addressed during residency [22–25]. These issues include (i) a relative lack of culturally understood communication skills, and (ii) disparities between the expected behaviour in Canadian medical culture versus that of the IMG’s country of training. Research by Pilotto et al. (2007) identified the following specific issues involving communication skills and cultural challenges in IMG training: adjustment to a culture with a more equitable doctor-patient relationship (and the associated difference in status); the high level of English language proficiency necessary; the importance of developing IMGs’ communication skills when working with patients; the importance that clinicians understand IMGs’ expectations about teaching and learning; and the need for IMGs to be able to interact with a wide variety of people [9].

In a study by Zulla et al. (2008), Residency Program Directors indicated that communication skills and professionalism were the main challenges faced by IMGs [26], a finding that, interestingly, was not shared by IMGs. In light of findings like this, we propose that it is imperative for IMGs and residency programs to identify underlying cultural discordances that lead to communication and professionalism problems in training and, eventually, in primary care.

Culture is often depicted as an iceberg: the small peak detectable to our senses is merely a glimpse of the large, submerged mass of values and thought processes that constitutes each culture [27]. In a clinical teaching environment the discernible manifestations of culture could include such concepts as communication, language, appearance, dress, procedures, organizational structure, and reporting. These concepts have all been explored in the medical education literature. However, it is the invisible part of culture that includes the concepts of teamwork, hierarchy, relationships and use of time – all elements of participation and access – and which cannot directly be observed that is of interest in this proposal, “the part of culture that needs to be inferred from what people say and do” [27]. These are the spheres in which we aim to identify important differences among residents in our programs.

Given the importance of IMGs in the Canadian health workforce and the difficulty of adjusting to cultural differences, an assessment tool that could identify hidden cultural drivers and subsequently guide educational programs would be beneficial. We identified an existing assessment tool that could be modified to suit medical education: the Organization and Culture Questionnaire (OCQ) [27]. This tool was developed by Dr. Lionel Laroche to support the better functioning of international business teams, and has been used successfully by many professionals in business and government to understand the impact of culture on individuals’ thinking and behaviours. Participants who complete the OCQ receive a customized report that compares the participant with the sample groups regarding various cultural difference issues. However, the OCQ has never been used to assess health professionals or residents in training, and therefore has not been psychometrically tested in these populations. This is a vital but currently missing component if the OCQ is to be used to assess the cultural vulnerabilities of IMGs.

The purpose of modifying the OCQ to create a new instrument, the ICDRE, was to gather self-reported information related to residents’ perceptions of their residency experience. The perceptions of interest included the concepts of hierarchy; use of time; relative importance of technical and soft skills; the preceptor-resident relationship; feedback; teamwork; problem-solving; and decision making. These perceptions formed the constructs of the ICDRE. This paper describes the creation and refinement of the ICDRE from the OCQ, the validation of the ICDRE questionnaire, and the application of the instrument in exploring cultural differences among CMGs and IMGs at the University of Ottawa.

### Theoretical framework

Medical residency can be understood as an instance of Lave and Wenger’s notion of situated learning [28–32]. Although Lave and Wenger maintain that all learning is situated, and thus that situated learning is not one *type* of learning amongst others, medical residency could be said to be the most canonical instance in the course of medical training, partly because it is so closely akin to apprenticeship [33, 34]. A key aspect of situated learning is the particular mode through which trainees can legitimately participate in the practice before they have become experts, a notion that Lave and Wenger term “legitimate peripheral participation”. Through participation, learners develop their identity *as* practitioners, i.e., in this case, physicians. Thus, barriers to participation, such as cultural differences in concepts of teamwork, hierarchy, and risk tolerance can have an impact on professional identity, as well. The question we address here with the ICDRE instrument, then, is to what extent cultural differences create barriers, particularly (but not exclusively) for IMGs.

### Methods

This study comprised four phases, in which the ICDRE was successively (i) refined and edited, (ii) tested, (iii) piloted, and finally (iv) deployed in a survey.

### Participants

Participants in this first phase of the pilot were expert preceptors (in addition to the investigators, ten expert faculty representing seven medical specialties) from the University of Ottawa, Faculty of Medicine, who have extensive experience practicing medicine in Canada and teaching residents including IMGs. Participants in the second phase were 11 volunteer residents. Participants in the third phase were all the members of the Family Medicine resident body (*N* = 140). Finally, participants in the fourth phase were members of a population comprising residents and faculty physicians from a wide variety of specialties across the entire Faculty of Medicine.

### Phases

Once the ICDRE had been refined, it was tested in a “think aloud” session to explore the volunteer residents’ use of the scale and interpretations of the items. The revised ICDRE was then piloted online to gather preliminary statistics on the scale. Participants were asked to indicate their level of agreement with each item using a 7-point Likert scale (1 = strongly disagree, 7 = strongly agree) with an option for “not applicable.” Next, the ICDRE questionnaire was validated using a framework based on modern validity theory [35–37]. This involved collecting data related to five sources of evidence for construct validity: content, response process, internal structure, relation to other variables, and consequences. For the purpose of the initial phase of our validation process we focused on collecting data from the first three sources. Once the questionnaire had been validated for use with family medicine residents, it was distributed electronically a year later to the survey population.

The study was reviewed and granted approval by the Ottawa Health Science Network and Bruyère Continuing Care research ethics boards (Protocol # 20150333-01H). All participants in the questionnaire study gave informed consent by active participation. All participants in the “think aloud” session gave informed consent by active participation in the session.

### Data collection 

#### Content evidence

Content evidence refers to the extent to which an instrument’s items represent the content of the concept of interest [38]. In our case, this means whether the items of ICDRE adequately measure residents’ perceptions of their residency experiences with regard to the concepts of: (i) use of time; (ii) sense of hierarchy; (iii) relative importance of technical and soft skills; (iv) the preceptor-resident relationship; (vi) feedback; (vii) teamwork; (viii) problem-solving; and (ix) decision making. Content evidence is subjective and therefore requires a “detailed description of steps taken to ensure that the items represent the construct” [38]. We used Squires’s process for collecting content evidence which involved: 1) a priori efforts by the scale developers to develop items that are based on the existing literature theory and 2) a posteriori efforts by using a panel of subject matter experts to evaluate the relevance of the scale’s items to the concept of interest [38].

A priori efforts: AE, DA, DS, and LL drew on their professional experiences as educators, previous work with foreign trained professionals, and the relevant recent literature on cultural competencies to develop items and create the modified OCQ (the ICDRE).

A posteriori efforts: A dinner meeting with the expert panel was held to help establish content validity of the ICDRE. Physicians from a variety of departments in the University of Ottawa Faculty of Medicine were recruited by AE. Prior to the meeting, the panel was sent the ICDRE from the a priori efforts to assess the readability of the questionnaire and to determine whether each item properly addressed a relevant aspect of the resident training experience.

#### Response processes

The next stage required several residents to review the ICDRE in a “think aloud” session. Each session was recorded and transcribed verbatim. Feedback was recorded regarding wording difficulties with questions, areas where intent of a question is unclear, and whether the questionnaire feels onerous to complete. Transcripts were examined by the investigators to identify areas in need of further amendment.

Following the “think aloud” session, the ICDRE was ready for testing with a larger group of family medicine residents. The data from this pilot survey were used to explore the internal structure of the ICDRE.

#### Internal structure and reliability

The third source of validity evidence, internal structure, refers to the psychometric characteristics of the scale properties. This requires exploring the ICDRE’s ability to be reproduced and generalized [36]. At this point, the ICDRE had been pared down to target three of the constructs known to influence teamwork (sense of hierarchy, individuality, and risk tolerance) [27] such that the questionnaire comprised three corresponding subscales. The internal consistency reliability of the ICDRE’s scales was assessed with Cronbach’s alpha and reported with 95% confidence interval (CI). Test-retest reliability was also calculated for participants who completed both the think-aloud session and the electronic pilot questionnaire. Item-total correlations were computed between individual item responses and respective scale scores. Items with low item-total correlations were discarded from the scale. This process was repeated in an iterative fashion until all scales displayed adequate internal consistency.

#### Implementation – participant feedback and consequences

After the completion of the pilot, the ICDRE was electronically distributed by the postgraduate office of the Faculty of Medicine and individual department administrators. Faculty and residents who completed the ICDRE received an individualized report of their results that showed how their responses compared to the means of CMGs, IMGs, faculty and the overall group.

## Results

### Pilot phase

The panel of expert preceptors reviewed the ICDRE and trimmed the instrument from 64 to 48 items. The instrument was refined in this way to eliminate redundant or irrelevant items. A total of 66 family medicine residents (47% response rate) completed the pilot ICDRE. Participant demographics are detailed in Table 1. Participants responded to questions grouped into the three subscales – hierarchy, individualism / teamwork, and risk tolerance – denoted as H, I, and R. These three subscales constituted the final ICDRE instrument.

**Table 1:** Demographics for ICDRE pilot

Type	CMG	42	63.6%
	IMG	15	22.7%
	IMG (CSA)	9	13.6%
Gender	Female	45	68.2%
	Male	21	31.8%
Age	20–29 years	13	19.7%
	30–39 years	42	63.6%
	40–49 years	9	13.6%
	50 years +	2	3.0%
Level	PGY 1	36	54.5%
	PGY 2	29	43.9%

Using the results from the family residents surveyed, we calculated the overall Cronbach’s alpha of the instrument to be 0.675 (CI: 0.540, 0.785) suggesting that the ICDRE has adequate internal consistency reliability. Individual subscales showed lower internal consistency reliability. The 24 *Hierarchy* items had internal consistency of α = 0.442 (CI: 0.210, 0.632); the 13 *Individualism/Teamwork* items α = 0.276 (CI: − 0.034, 0.525); and the 11 *Risk Tolerance* items α = 0.330 (CI: 0.043, 0.560). We assessed test-retest reliability among seven participants who completed both the think-aloud session and the electronic pilot questionnaire using total numerical scores at each time point. We calculated a Pearson’s coefficient of 0.821 (*p* = 0.024), which suggests considerable correlation between time points of the questionnaire.

Item total correlations yielded a number of correlations, both positive and negative, for each subscale. For the *Hierarchy* subscale, 41 statistically significant correlations occurred between items, with a range in absolute value of the correlation coefficient (r) of 0.261–0.600. For *Individualism/Teamwork* we observed eight correlations ranging in absolute value of 0.263–0.698. There were three correlations on the *Risk Tolerance* subscale, ranging from 0.260–0.442. Very few of these correlation coefficients had an absolute value greater than 0.5 (*n* = 3 for Hierarchy, *n* = 2 for Individualism/Teamwork, *n* = 0 for Risk Tolerance), suggesting that there was no redundancy among items in the instrument. ICDRE items are included as supplementary material (see Additional file 1).

### Implementation group

Following the instrument validation with family medicine residents, the ICDRE was administered to residents and faculty from several specialties. We received responses from 86 residents and 14 faculty physicians, representing 30 different specialties. Demographic information for the implementation group is reported in Table 2.

**Table 2:** Demographics for 2015 implementation group

Type	CMG	83	83.0%
	IMG	10	10.0%
	IMG (CSA)	7	7.0%
Gender	Female	64	64.0%
	Male	36	36.0%
Age	20–29 years	41	41.0%
	30–39 years	43	43.0%
	40–49 years	7	7.0%
	50 years +	8	8.0%
Level	PGY 1	30	30.0%
	PGY 2	17	17.0%
	PGY 3	10	10.0%
	PGY 4	6	6.0%
	PGY 5	9	9.0%
	Fellowship	9	9.0%
	Faculty	14	14.0%

IMGs and CMGs were identified through a survey question where respondents were asked to identify “in which country did you complete medical school?” All respondents identifying a country other than Canada were coded as IMG. The IMG group represented a wide range of medical graduates trained in Australia, South, East and Western Asia, North Africa, South America, South, Central and Eastern Europe and North America. Comparing IMGs to CMGs in our implementation group using 2-tailed t-tests yielded seven items for which the two groups significantly differed (Table 3). Though statistically significant, none of these differences are drastic, and the occurrence of a difference in only seven of the 48 items suggests that the two populations do not have vastly different perspectives on hierarchy, individualism/teamwork, or risk tolerance.

**Table 3:** Differences between IMG and CMG Groups

Item	CMG Mean	IMG Mean	*p* value
The main reason for having a team structure is so that everyone knows who has authority over whom (H2)	2.23	3.13	0.032
When my staff physician points out one of my weaknesses it gives me a chance to correct it (H11)	6.57	5.87	0.008
To be promoted from PGY 1 to PGY 2 one needs to take on more responsibility (H12)	6.17	5.47	0.015
Faculty should use their influence to get a residency position for one of their relatives or friends (H23)	1.17	1.73	0.022
A team is more effective when each team member focuses on their own tasks and responsibilities (I8)	4.68	5.53	0.048
I plan my vacations well in advance (R7)	5.57	4.67	0.040
The most effective way to manage a clinical problem is to consider a number of possible interventions (R8)	6.04	5.20	0.012

While investigating frequencies for individual questions, we identified interesting outlier cases in which one participant had a strong opinion that differed from all the others. Table 4 lists selected examples. The occurrence of such strong, atypical opinions among IMGs, CMGs, and CSAs reveals a diversity of opinions within each group. Unusual responses to certain ICDRE items may be more indicative of personality differences or other hidden factors that are not necessarily related to cultural background.

**Table 4:** Cases of divergent opinions

Item	Response	Type of Grad.	Med School Country	Years in Canada	Gender	Level
When doctors make mistakes and admit it to their team they lose credibility and undermine staff confidence in their judgment (H6)	Strongly Agree	IMG	Egypt	10	Female	Fellowship
When my staff physician points out one of my weaknesses it gives me a chance to correct it (H11)	Strongly Disagree	IMG	Egypt	10	Female	Fellowship
I prefer working with staff doctors who give me enough freedom so that I can determine the best direction for my learning (H18)	Strongly Disagree	IMG	Egypt	10	Female	Fellowship
When doctors make mistakes and admit it to their team they gain the respect of their team for their honesty (H9)	Strongly Disagree	CMG	Canada	32	Male	PGY 5
I regularly ask my staff physician about my progress (H14)	Strongly Disagree	IMG (CSA)	Australia	39	Male	PGY 4
I prefer working in clinical settings where credit is given to individuals not teams (I5)	Strongly Agree	CMG	Canada	27	Male	PGY 2

## Discussion

The IDCRE questionnaire bridges a critical gap in communication between residents and clinical preceptors that has been widely recognized in many medical programs [39–41]. Closing it can help to ensure better integration and success for IMGs, as doing so increases their access to medical practitioners and mature medical practice. It can also aid in minimizing downstream disruption to patient care, which results when expectations of professional demeanor differ within healthcare teams [40]. Our questionnaire yielded few statistically significant differences between CMGs and IMGs; those that did arise were subtle in nature. The binary comparison of “Canadian” vs. “non-Canadian” assumes a level of homogeneity within each group that may not be present in reality. Such a comparison fails to consider the great diversity in ethnic and cultural background among Canadian nationals and also assumes a standard Canadian culture across the country. Furthermore, the IMG group represented a wide range of home countries; medical graduates trained in Bangladesh, Russia, and Iran cannot be expected to have experienced the same culture while in medical school. Indeed, such diversity within groups is evident in the highlighted outliers, where individuals from each group expressed strong opinions not in line with the rest of the group. More culture-specific information could be gleaned using a more granular approach to the IMG group, perhaps by subdividing into geographical regions, but this approach may lead to small sample sizes and stereotyping.

Another factor to consider is the possible presence of CSAs among the IMG population. These are Canadian citizens who attended medical school in another country. Of the 23 IMG respondents, 26.1% were identified to be CSA (*n* = 6), which was derived from the survey question “in which country were you born?” It is likely that the cultural beliefs held by such individuals are already ingrained before they left Canada, and so even though they are technically IMGs, their cultural beliefs may more closely reflect those of Canada than the country in which they obtained their MD degree. At the same time, being exposed to the medical culture of a different country is also likely to influence graduates’ beliefs within the medical context.

Clinical teachers acknowledge the need for explicit and implicit training in cultural competence [39], but diversity training in medical education programs falls short of recommended standards [41]. Jha et al. (2015) present several case studies on the challenges that arise when healthcare teams include different cultural backgrounds [40]. These differences are often taken for granted, but can lead to disruptions in confidentiality, patient rights and patient care if they are not identified and discussed. Ideally, a medical program would offer a comprehensive curriculum, ongoing support, and cultural awareness at the organisational and training levels. In Canadian medical schools, the inclusion of a cultural diversity education component has been mandatory since 2002 [42]. However 67% of these schools (11 out of 16) have indicated that their existing curriculum is inadequate [42–44]. Introducing curriculum changes can be challenging and time consuming [45, 46]. Administration of the ICDRE might help to achieve levels of awareness similar to those of a formalized education program.

If administered during the resident selection process, the ICDRE could help to identify areas that may be a challenge to the resident with regard to hierarchy, individualism, or risk tolerance. If the resident and his/her preceptor is comfortable doing so, any areas of difficulty could be discussed at the beginning of the residency to minimize friction. The questionnaire could also be used as an interventional tool with existing residents, not just incoming ones, as it provides discrete items related to professionalism on which residents are scored and compared to their peers. Preceptors can identify areas in which residents’ beliefs vary considerably from the local medical culture or from their peers, and coach the resident accordingly. At the institutional level, aggregate data from this instrument could be used to inform and shape the postgraduate curriculum. If many members of an incoming resident cohort have difficulty taking criticism without feeling humiliated, for example, the program could consider whether the currently used avenues for feedback to residents are tailored adequately for criticism to be received constructively.

This study has a number of limitations. First, binary analysis of data between the “Canadian” and “non-Canadian” groups does not capture the nuances of identity that exist within these population groups. Increasing the sample size and finding ways to stratify the population with more granularity may help to pick up on further differences between the two populations. Competing demands of medical school curriculums might make it difficult to incorporate the questionnaire; logistically finding a way to incorporate it into the curriculum might not be feasible if time does not allow.

Another limitation is the self-reporting by residents and preceptors. Residents may be reluctant to acknowledge their own biases, preferring to answer questions in a way that aligns more with the beliefs of their preceptors. Furthermore, medical professionals may not be aware of their behaviour regarding hierarchy, individuality versus teamwork, and risk tolerance, and answers on the ICDRE tool might differ from their true professional behaviour. Third, this tool was validated entirely at one academic centre, with a sample of 68 residents. In order to increase the generalizability of the results, the tool must be administered in other education settings. Although the sample of 68 residents may be considered small for this type of analysis, it should suffice to establish the internal consistency of the instrument prior to its use with larger populations. The Cronbach’s alpha of 0.675, though lower than ideal, is generally considered acceptable for early stages of research [47]. Increasing the sample population might help increase the internal consistency reliability. Finally, hierarchy, individuality, and risk tolerance are only three areas within professionalism and cultural differences. Laroche and Yang (2014) have identified three other factors that should be considered: one’s technical and intrinsic skills; one’s understanding of cross-cultural feedback; and finally one’s ability to communicate cross-culturally [48].

## Conclusions

The ICDRE shows promise as a measurement of cultural beliefs among residents, with a reasonable level of internal consistency. Analysis of a resident population in this study yielded few significant differences between the CMG and IMG groups as a whole, but highlighted a diversity of opinions within the resident body. The instrument may also be useful in graduate medical education as a means to detect potential problem areas for incoming residents or as an interventional tool for preceptors to assess residents’ professionalism.

## Supplementary Information

**Additional file 1.**

## Data Availability

The data used and/or analysed during the current study are available from the corresponding author on reasonable request.

## Abbreviations

CSA: Canadian Studying Abroad
CMG: Canadian Medical Graduate
FMG: Foreign Medical Graduate
ICDRE: Impact of Cultural Differences on Residency Experiences
IMG: International Medical Graduates
OCQ: Organization and Culture Questionnaire
