# Supplementary material for: Capturing the impact of cultural differences in residency
Source: BMC Med Educ. 2021 Feb 18;21:115. doi: 10.1186/s12909-021-02548-4 (PMC7890890; doi:10.1186/s12909-021-02548-4)
Supplement: Supplementary file 1 — Additional file 1. [file 12909_2021_2548_MOESM1_ESM.docx]

Supplementary Material

The ICDRE Questionnaire

HIERARCY

H1_I prefer my staff physician follows my progress closely and provide advice and encouragement

H2_The main reason for having a team structure is so that everyone knows who has authority over whom

H3_When I realize that one of my patients is making a poor and potentially costly health decision, such as smoking, I would feel it my responsibility to discuss the issue with my patient

H4_When my staff physician points out one of my weaknesses it makes me feel humiliated/lose face

H5_If my staff physician criticized me in front of others I would find it difficult to face the team the following day

H6_When doctors make mistakes and admit it to their team they lose credibility and undermine staff confidence in their judgment

H7_If my staff physician criticized me in front of others I would be determined to show everyone I was better than they think

H8_I prefer that my staff physician gives me freedom to make decisions on my own

H9_When doctors make mistakes and admit it to their team they gain the respect of their team for their honesty

H10_A senior nurse should be paid more than a junior resident

H11_When my staff physician points out one of my weaknesses it gives me a chance to correct it

H12_To be promoted from PGY 1 to PGY 2 one needs to take on more responsibility

H13_The main reason for having a team structure is so that everyone knows who is expected to do what

H14_I regularly ask my staff physician about my progress

H15_A team structure in which there are multiple staff physicians is desirable because it helps balance patient needs

H16_To be promoted from PGY 1 to PGY 2 one needs to attain defined competencies

H17_A good doctor should have precise answers to the questions that the team raises about their clinical decisions

H18_I prefer working with staff doctors who give me enough freedom so that I can determine the best direction for my learning

H19_A team structure in which there are multiple staff physicians is undesirable because it leads to confusion

H20_In my experience, residents are hesitant to express disagreement with their staff physicians

H21_When a resident has a weakness, his/her staff physician should point it out

H22_I prefer learning from staff physicians who give me clear directions so that I know what they want me to learn

H23_Faculty should use their influence to get a residency position for one of their relatives or friends

H24_When doctors make mistakes they should admit it

INDIVIDUALISM/TEAMWORK

I1_The main criteria for promotion in a hospital should be accomplishments and performance

I2_The main criteria for promotion in a hospital should be loyalty to the organization and seniority

I3_Decisions made by groups are usually better than decisions made by individuals

I4_I prefer working in clinical settings where credit is given to teams, not individuals

I5_I prefer working in clinical settings where credit is given to individuals not teams

I6_A team is more effective when team members provide information and support to one another

I7_People are more effective when they focus on one task or project at a time

I8_A team is more effective when each team member focuses on their own tasks and responsibilities

I9_I prefer working in a clinical setting where people work individually

I10_I prefer working in a clinical setting where people work in teams

I11_When my contribution to the team leads the team to make a flawed decision the responsibility is mine

I12_When I work in a team and things don’t work out well there is no individual blame

I13_People are more effective when they work on several tasks or projects in parallel

RISK TOLERANCE

R1_A good solution is a solution that I understand in detail so that I can reapply it widely and easily

R2_Doctors who make decisions slowly compromise patient outcomes

R3_A good solution is a solution that works even though I do not fully understand why

R4_If I am finding something difficult I will ask for help

R5_Doctors who make decisions quickly make the wrong decisions

R6_If team members don’t stick to the rules, the team can descend into chaos

R7_I plan my vacations well in advance

R8_The most effective way to manage a clinical problem is to consider a number of possible interventions

R9_The most effective way to manage a clinical problem is to analyze all the data in detail before considering any intervention

R10_I tend to prescribe drugs that have been introduced recently

R11_I tend to prescribe drugs that have an established track record
